# Knowledge, Attitudes, and Practices of Dermatologists on Chronic Pruritus and Its Management

**DOI:** 10.7759/cureus.93494

**Published:** 2025-09-29

**Authors:** Ankur Talwar, Shikha Shivhare, Devesh K Joshi, Monil Gala, Snehal S Muchhala, Seema V Bhagat, Arti Sanghavi, Sagar Katare, Bhavesh P Kotak

**Affiliations:** 1 Dermatology, Talwar Skin Beauty and Laser Institute, Lucknow, IND; 2 Dermatology, People's College of Medical Sciences and Research Centre, Bhopal, IND; 3 Medical Affairs, Dr. Reddy's Laboratories Ltd., Hyderabad, IND

**Keywords:** antihistamines, chronic pruritus, dermatologists, hydroxyzine, hydroxyzine sustained-release formulation

## Abstract

Background

Chronic pruritus (CP) is a prevalent and distressing condition with a significant impact on patients' quality of life. Despite advances in understanding its pathophysiology, management remains challenging, necessitating insights into dermatologists' perspectives on CP treatment.

Objective

This study aimed to assess the knowledge, attitudes, and practices (KAP) of dermatologists regarding CP management, with a focus on antihistamine use.

Methods

A cross-sectional study was conducted among dermatologists across India. A total of 220 participants completed the quantitative KAP questionnaire, while 15 (6.82%) underwent qualitative interviews. Data were analyzed using descriptive statistics and thematic analysis.

Results

Dermatologists showed good knowledge of CP pathophysiology, with histamine and H1 receptors recognized as key mediators. Antihistamines were widely used, and many respondents reported a shift toward once-daily regimens to improve symptom control and adherence. Patient education was identified as a critical need, alongside further safety and efficacy data in high-risk groups.

Conclusion

Dermatologists emphasized a patient-centered approach, prioritizing the identification of underlying causes while addressing symptoms. Hydroxyzine and its sustained-release formulation were regarded as convenient and effective, supporting simplified dosing and better adherence. Strengthening patient education remains essential to optimizing CP management.

## Introduction

Pruritus or itch can be defined as a sensation that provokes the desire to scratch. Scratch can also be accompanied by rub, pinch, or even the use of various devices to help soothe the itching [1]. Chronic itch is a common and distressing symptom that arises from a variety of skin conditions and systemic diseases [2]. The International Forum for the Study of Itch (IFSI) defines chronic pruritus (CP) as pruritus lasting six weeks or longer [3]. Pruritus can occasionally precede the clinical diagnosis of systemic diseases by several years, a phenomenon described as premonitory pruritus [1]. Omidvari et al. reported a case of a 16-year-old with generalized pruritus persisting for approximately four years before the diagnosis of Hodgkin's disease; the symptom resolved after the first round of chemotherapy [4]. This is of clinical importance, as pruritus has also been documented as a paraneoplastic manifestation, occurring in up to 30% of patients with Hodgkin's lymphoma, where it may present as one of the earliest clinical signs [5]. The cause of chronic itch is historically classified into four categories of itch: dermatologic (associated with primary skin diseases, such as atopic dermatitis and psoriasis), neuropathic (associated with nerve fibers, such as brachioradial pruritus and small-fiber polyneuropathy), psychogenic (associated with psychiatric conditions, such as generalized anxiety disorder and delusions of parasitosis), and systemic (associated with conditions such as end-stage renal disease and cholestasis) [6]. Some advances have been made in understanding the pathophysiology of CP; however, despite being a common symptom of numerous skin diseases, its underlying mechanisms remain unclear. CP develops through a complex interplay of immune dysregulation, neuronal sensitization, and altered central nervous system (CNS) processing, leading to persistent and difficult-to-treat itching [7].

Global evidence highlights the significant burden of CP. A German population-based cross-sectional study reported a point prevalence of CP at 13.5%, a 12-month prevalence of 16.4%, and a lifetime prevalence of 22% [8]. A follow-up study further found a 12-month cumulative incidence of CP to be 7%, with lifetime prevalence increasing to 25.5% [9]. A more recent international study highlighted the global impact of pruritus, reporting a worldwide prevalence of 39.8%, with the highest prevalence (43.3%) observed in individuals aged 65 years and older [10]. In the Indian setting, there are insufficient data reported on the prevalence and epidemiology of CP in the general population [11]. In a study of 120 maintenance hemodialysis patients, 55.8% reported experiencing uremic pruritus [12]. In a cross-sectional knowledge, attitudes, and practices (KAP) study with physicians and diabetes patients, Kalra et al. reported 14% of patients with diabetes experienced pruritus without visible skin lesions [13]. The factors such as eczema, dry skin, asthma, liver disease, high body mass index, and anxiety scores have been identified as key determinants of CP [9].

CP significantly impacts patients' quality of life, leading to sleep disturbances, anxiety, depression, and social withdrawal. Psychological factors can also contribute to CP and scratching behavior, affecting both skin-related and non-skin-related conditions [14]. Anti-inflammatory agents, such as antihistamines and steroids, play a crucial role in managing CP, particularly in cases related to cutaneous inflammatory and allergic disorders. First-generation sedative antihistamines, especially hydroxyzine, are the most effective, and H1-type medications have been studied the most among antihistamines. In addition to binding to H1 receptors, first-generation antihistamines also interact with muscarinic, alpha, dopamine, and serotonin receptors. This results in a sedative action that improves sleep, which is frequently disturbed in CP, and reduces deficits in quality of life and allergic conditions [11].

CP is one of the most problematic symptoms in dermatological practice, severely affecting quality of life and mental well-being. While patient-reported outcomes in CP have been widely studied, there is limited global and country-specific evidence on medical practitioners' perspectives regarding its management. In particular, data from India are scarce, despite the high burden of dermatological disease. This study was therefore undertaken to assess the KAP of dermatologists regarding CP.

## Materials and methods

Study design

This was a cross-sectional, mixed-methods (quantitative and qualitative), question-based study conducted among practicing dermatologists selected randomly from across India. The inclusion criteria required dermatologists to have a minimum of five years of clinical experience and be actively treating CP cases in various healthcare settings, including clinics, hospitals, and medical colleges. Dermatologists unwilling to voluntarily participate in the study and/or provide written consent were excluded. The study included a total of 220 dermatologists for the quantitative part, out of which 15 (6.82%) participants took part in qualitative interviews as well.

Sample size calculation

Cochran's formula ($n_0=Z^2 p(1-p)/d^2; n=n_0/[1+(n_0-1)/N]$), as applied in similar KAP studies, was used to estimate the required sample size for the current study [15,16]. Assuming a total of approximately 12,500 registered dermatologists in India, a 95% confidence level, a 6% margin of error (d=0.06), and an expected proportion of p=0.70, the calculated sample size was 221 participants.

Questionnaire development

A quantitative KAP questionnaire was formulated covering all three subsections of Knowledge, Attitudes, and Practices to gauge the participants on these three categories, based on the information available in the literature. Another qualitative questionnaire was also formed to capture the opinions and practices of dermatologists regarding CP and its management. The questionnaires used in our study are not formally validated but were adapted from previously published tools with demonstrated reliability in similar contexts. The complete questionnaire can be found in the Appendices.

Data collection

Two authors were trained to collect, categorize, and tabulate data. They coordinated with participants, addressed the issues of non-responders, and conducted interviews with selected respondents for the qualitative part.

Data in the quantitative phase was collected via online (Google Forms, Google LLC, Mountain View, California, United States) and offline (pen-paper) modes. Participants provided demographic details, including age, gender, practice location, affiliation, experience, and CP case management. The questionnaire was not structured to show the subdomain of KAP to the participants to minimize bias. For qualitative assessment, selected dermatologists participated in physical or virtual interviews. They first completed the objective questionnaire, followed by an open-ended interview, with responses audio-recorded, transcribed, and archived for analysis.

Data analysis

The data obtained through Google Forms and offline questionnaires were tabulated in an Excel spreadsheet (Microsoft Corporation, Redmond, Washington, United States), and a thorough quality check was performed on the tabulated data, where a manual cross-check was done between random responses to ensure reliability. All continuous variables were expressed as mean±SD, while qualitative variables were expressed as proportions or percentages. Frequency tables were created for all questions to depict the frequency of each response among respondents. For qualitative data, interview recordings were transcribed, categorized, and analyzed for key findings/insights. These insights, along with the verbatim responses to support them, were listed to understand the outlook of participants towards CP and its management.

Ethical considerations

The study was approved by the Independent Ethics Committee of Shubhdisha Business Solutions (approval number: DRL-IND-GGI-056-ATAR/2024) and was registered on the Clinical Trials Registry-India (CTRI) (CTRI number: CTRI/2024/04/066054; date: 22/04/2024). Informed written consent was obtained from participants prior to filling out the questionnaire. The participants were fairly compensated for their time.

## Results

Participants' sociodemographic distribution

Among the 220 participants, 114 (52%) were male, whereas 106 (48%) were female from 17 states across India. Fifteen (6.82%) respondents were younger than 30 years, and 13 (5.91%) were older than 60 years. Most of the respondents were from the age group of 31-40 years (106; 48.18%), followed by 41-50 years (66; 30%). The mean age of the participants was 41.48±10.4 years, and the median age was 40 years. The clinical experience of the participants ranged from a minimum of five years to a maximum of 51 years, with most participants having the clinical experience of 5-10 years (108; 49.09%), followed by 11-20 years (72; 32.73%). The mean clinical experience of the respondents was 14.21±9.48 years, while the median experience was 11 years. A sizable proportion of the participants (86; 39.09%) dealt with more than 20 cases of CP on a weekly basis. Out of the 220 participants, 102 (46.36%) dealt with 11-20 cases of CP, whereas 32 (14.55%) participants saw fewer than 10 cases per week (Table 1).

**Table 1:** Demographic distribution of participants

Sociodemographic characteristics	n (%) or mean±SD
Gender (male)	114 (52%)
Age (years)	41.49±10.41
Clinical experience (years)	14.21±9.48
Weekly chronic pruritus cases	
<10 cases	32 (14.55%)
10-20 cases	102 (46.36%)
>20 cases	86 (39.09%)

Participants' knowledge about CP and its management

Understanding of the Participants About CP and Its Classification

Among the 220 respondents, the majority (210; 95.45%) correctly identified more than six weeks cut-off for the categorization of pruritus as chronic. Participants also had good knowledge about the IFSI classification of pruritus, as 190 (86.36%) accurately classified Group I pruritus, while 169 (76.82%) correctly identified Group II pruritus.

Knowledge About the Common Causes and Etiology of CP

Dry skin was identified as the most common cause of pruritus by most of the participants (148; 67.27%), followed by eczema (62; 28.18%), allergies (57; 25.9%), and infections (16; 7.27%). Furthermore, 217 (98.64%) agreed that atopic dermatitis is the most common cause of CP in children. Additionally, 211 (95.91%) participants agreed that the H1 receptor and histamines play a critical role in the induction of CP.

Problems Associated With CP

**Figure 1:**
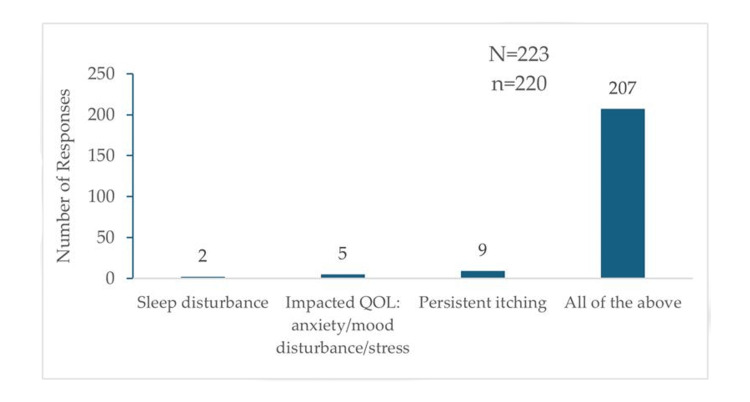
Problems associated with chronic pruritus as reported by the 220 participants "N" indicates the total number of responses, and "n" indicates the total respondents for this question. QOL: quality of life

Among 220 participants, nine (4.09%) identified persistent itching as the primary problem associated with CP, two (0.9%) reported sleep disturbance, and five (2.27%) noted a decrease in quality of life. However, most respondents (207; 94.09%) observed all three problems, i.e., persistent itching, sleep disturbances, and reduced quality of life, in their clinical practice (Figure 1).

Antihistamines and Their Role in the Management of CP

Most dermatologists recognized the role of antihistamines in the management of CP. Out of the 220 participants, 214 (97.27%) considered oral antihistamines to be the first-line treatment for this condition. Furthermore, 211 (95.91%) endorsed the use of antihistamines even when the cause of pruritus was not clear or not yet diagnosed. Additionally, 218 (99.09%) respondents supported incorporating antihistamines into the treatment plan for managing itch in cases where more than one pathology is associated with CP. A total of 218 (99.09%) agreed that hydroxyzine can be used as an effective management option for CP, while 214 (97.27%) acknowledged its anxiolytic benefits in addition to its anti-pruritic effects. Two hundred and fourteen (97.27%) participants agreed that uniform drug plasma concentrations over a prolonged period in the case of the sustained-release formulation of hydroxyzine helps in reducing the dosage frequency.

Participants' attitudes towards CP and its management

Perception Towards Problems Associated With CP

Out of the 220 participants, 217 (98.64%) believed that CP negatively affects patients' quality of life. Additionally, 218 (99.09%) participants agreed that CP causes sleep disturbance and anxiety. The majority of participants (216; 98.18%) emphasized that the management of CP should not solely target the underlying condition but should also address itching and associated anxiety.

Hydroxyzine as an Option for the Management of CP

Among the 220 respondents, 216 (98.18%) responding dermatologists perceived hydroxyzine to be safe for the management of CP. Furthermore, 216 (98.18%) participants recognized it to be better for the management of CP due to its additional anxiolytic properties apart from anti-pruritic action.

Perception Regarding the Sustained-Release Formulation of Hydroxyzine

Most of the participants (214; 97.27%) considered the sustained-release formulation of hydroxyzine to be beneficial in CP management, with 201 (91.36%) believing that this formulation has superior bioavailability compared to the immediate-release formulation of hydroxyzine. Additionally, 201 (91.36%) participants felt that the sustained-release formulation may reduce the need for up-dosing, leading to better patient tolerance and improved adherence to treatment.

Practices followed by participants for the management of CP

Choice of Drug for the Management of CP

**Figure 2:**
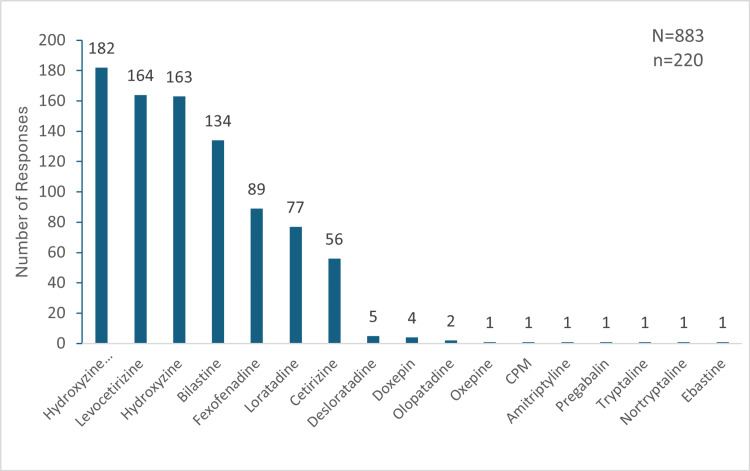
Prescription numbers of drugs for the management of chronic pruritus as reported by the 220 respondents "N" indicates the total number of responses, and "n" indicates the total respondents for this question. CPM: chlorpheniramine maleate

A total of 181 (82.28%) participants reported prescribing H1 antihistamines for more than 50% of the cases, whereas 83 (37.73%) prescribed them in almost all the cases, with a few exceptions. Out of the 220 participants, hydroxyzine was frequently used as a first-line treatment by 191 (86.81%), with 32 (14.55%) always prescribing it as their primary option. Sustained-release hydroxyzine was prescribed by 206 (93.64%) participants for CP, and 136 (66.02%) of these 206 participants used it for acute pruritus. Overall, sustained-release hydroxyzine, levocetirizine, hydroxyzine, bilastine, fexofenadine, loratadine, and cetirizine were the frequently used molecules for the management of CP (Figure 2).

Factors Responsible for Treatment Choice

**Figure 3:**
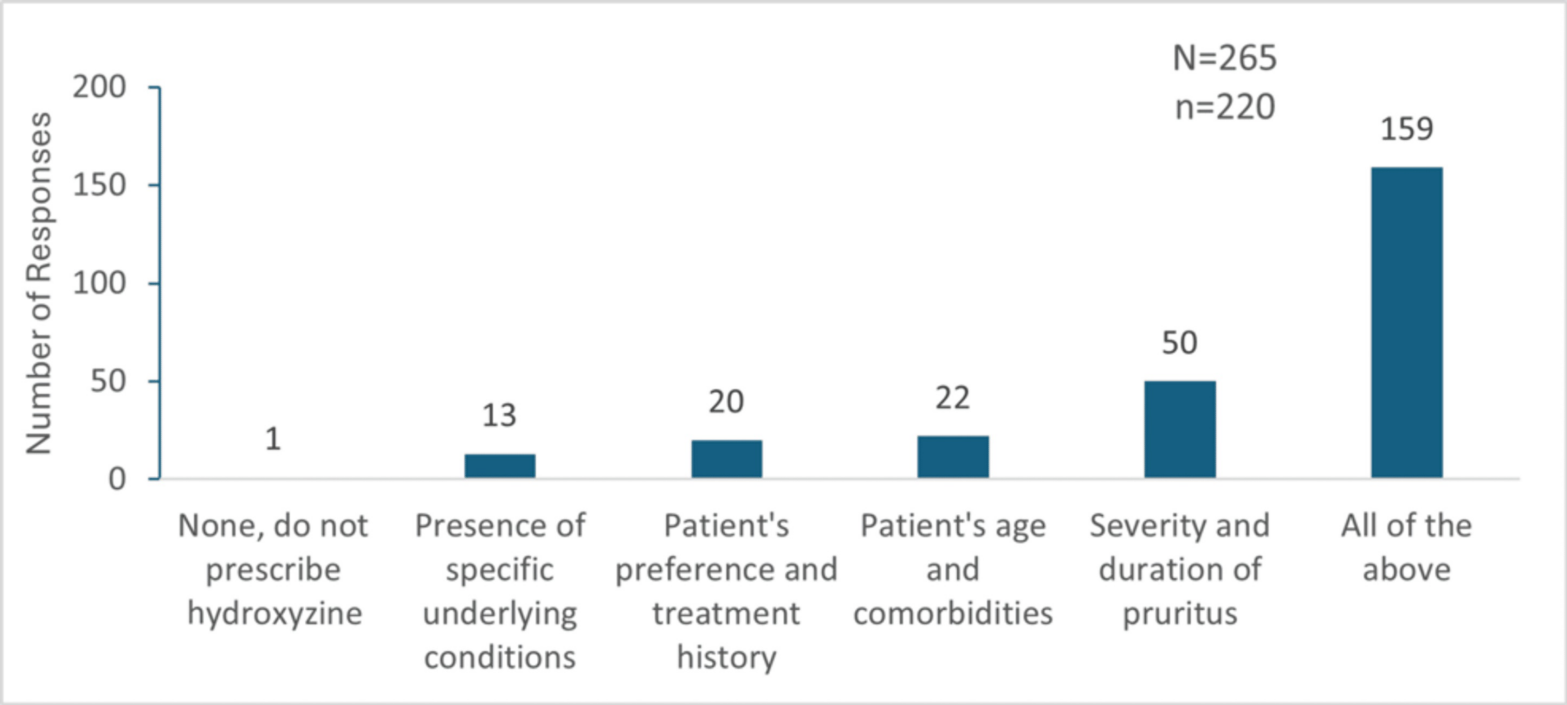
Factors considered by the participants while selecting hydroxyzine as the preferred drug for the management of chronic pruritus "N" indicates the total number of responses, and "n" indicates the total respondents for this question.

The severity and duration of pruritus were considered as the most important factors influencing their choice of hydroxyzine over other H1 antihistamines by 50 (22.72%) participants. Patient age and comorbidities were key for 22 (10%) participants, while 20 (9.09%) prioritized patient preference and treatment history. Specific underlying conditions influenced the choice for 13 (5.9%) participants. Notably, 159 (72.27%) participants considered all of these factors when selecting a treatment regimen for CP management (Figure 3).

**Figure 4:**
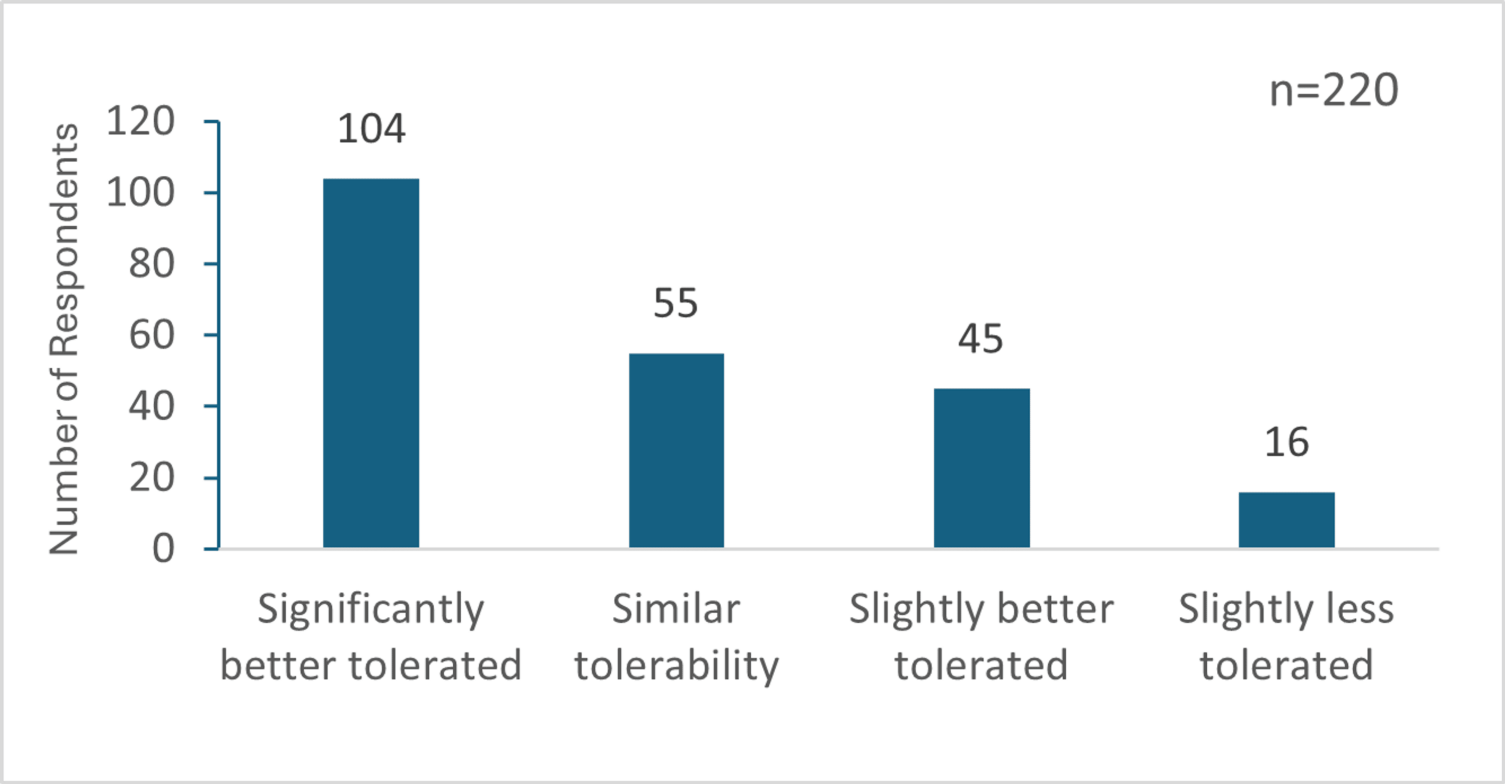
Tolerability of hydroxyzine compared to other H1 antihistamines in participants' practice "n" indicates the total respondents for this question.

Regarding hydroxyzine's effectiveness, 180 (81.82%) participants reported it as very effective for CP, and 39 (17.73%) considered it moderately effective. Additionally, 149 respondents found hydroxyzine better tolerated than other H1 antihistamines, while 55 considered its tolerability similar (Figure 4).

**Figure 5:**
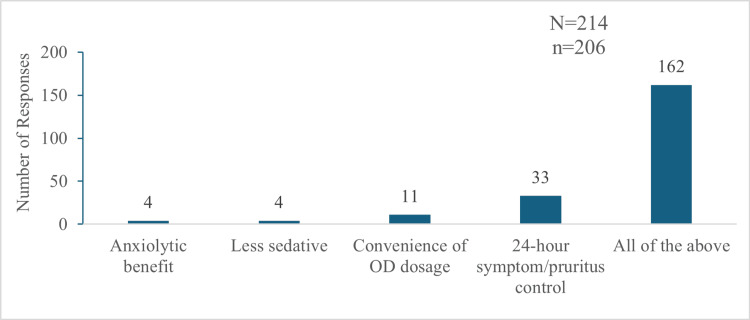
Benefits of the sustained-release formulation of hydroxyzine over the immediate-release formulation "N" indicates the total number of responses, and "n" indicates the total respondents for this question. OD: once-daily

Of the 206 participants prescribing sustained-release hydroxyzine, 197 (95.63%) reported it to be more effective than the immediate-release formulation, and 152 (73.79%) perceived it as less sedating. When asked about key benefits, 33 (16.01%) participants chose 24-hour symptom/pruritus control, 11 (5.33%) participants selected convenience of once-daily (OD) dosing, and four (1.94%) participants selected lesser sedation and anxiolytic benefit, each. The majority, 162 (78.64%) participants, reported all of these as the benefits of using the sustained-release formulation over the immediate-release formulation (Figure 5).

Treatment Plan for the Management of CP

**Figure 6:**
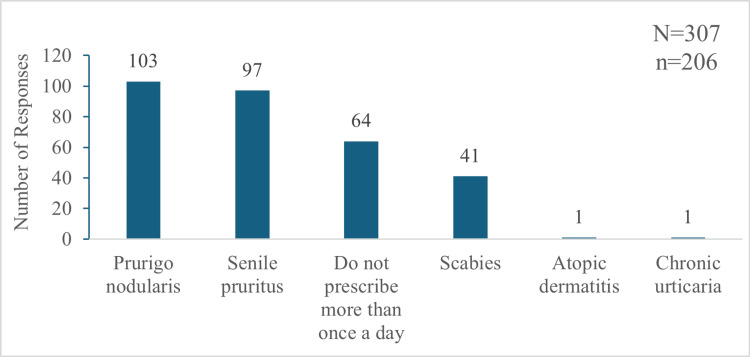
Indications of chronic pruritus for which hydroxyzine is prescribed more than once a day by participants "N" indicates the total number of responses, and "n" indicates the total respondents for this question.

**Figure 7:**
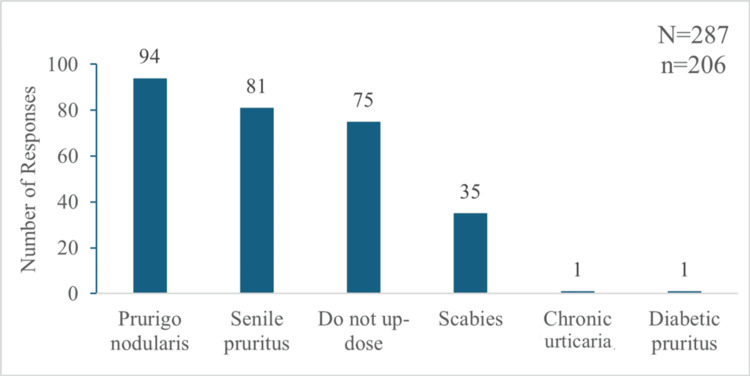
Indications of chronic pruritus for which participants up-dosed the sustained-release formulation of hydroxyzine "N" indicates the total number of responses, and "n" indicates the total respondents for this question.

Among the 220 participants, 178 (80.9%) prescribed hydroxyzine OD, 31 (14.09%) prescribed it twice daily (BID), and one (0.45%) used a thrice-daily (TDS) schedule. Additionally, nine (4.09%) used both once- and twice-daily regimens, and one (0.45%) used both BID and TDS dosing of hydroxyzine. In contrast, sustained-release hydroxyzine was predominantly prescribed OD by 194 (94.17%) of the 206 participants, with only seven (3.39%) opting for exclusively BID dosing, and five (2.43%) used both OD and BID doses. Conditions necessitating the up-dosing of hydroxyzine included prurigo nodularis, senile pruritus, and scabies. However, 64 (31.06%) out of these 206 respondents never increased the dosing frequency of hydroxyzine, and 75 (36.4%) never up-dosed sustained-release hydroxyzine for any indication (Figures 6-7).

Treatment Options Followed Prior to Using the Sustained-Release Formulation of Hydroxyzine

**Figure 8:**
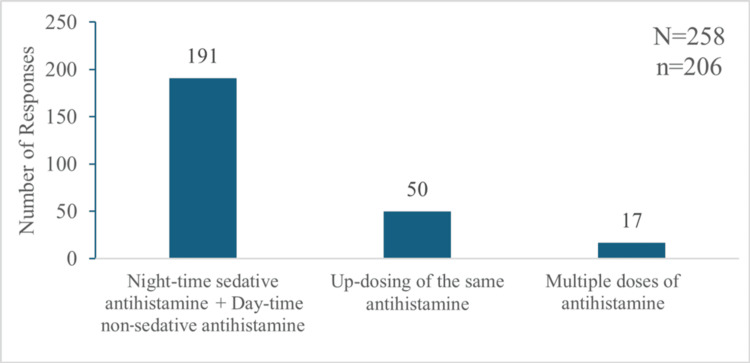
Treatment regimen followed by participants prior to using the sustained-release formulation of hydroxyzine "N" indicates the total number of responses, and "n" indicates the total respondents for this question.

For the respondents who have started using the sustained-release formulation (n=206), the earlier treatment regimen included using a sedative antihistamine at night and a non-sedative antihistamine in the morning, up-dosing of the same antihistamine, and multiple doses of the same antihistamine (Figure 8).

Treatment Regimen Followed by Participants Who Do Not Use the Sustained-Release Formulation of Hydroxyzine

**Figure 9:**
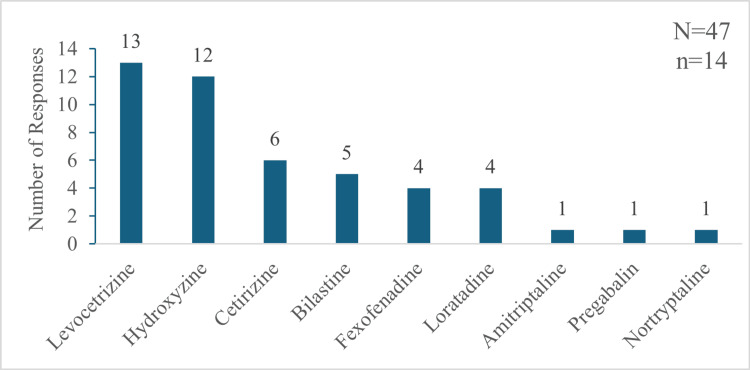
Drugs prescribed for the management of chronic pruritus by the participants who do not use sustained-release hydroxyzine "N" indicates the total number of responses, and "n" indicates the total respondents for this question.

In the case of participants who did not use sustained-release formulation (n=14), levocetirizine and hydroxyzine were the most common prescribed agents, followed by other H1 antihistamines and other anti-psychotic/epileptic drugs (Figure 9).

**Figure 10:**
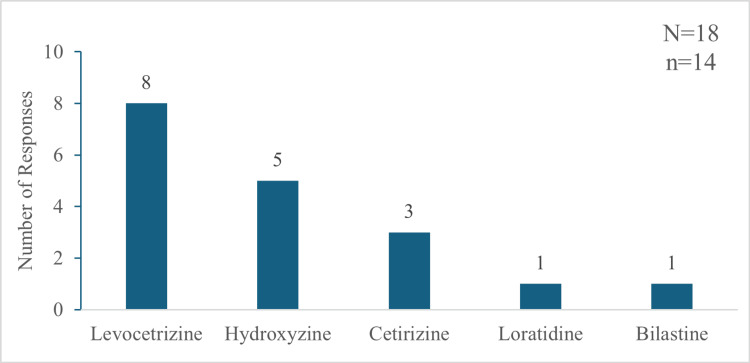
Current drugs that the non-prescribers of sustained-release hydroxyzine will replace for prescribing it "N" indicates the total number of responses, and "n" indicates the total respondents for this question.

When asked which agent they would replace if using sustained-release hydroxyzine, these 14 respondents named levocetirizine, hydroxyzine, and cetirizine as the drugs they would swap from their current regimen (Figure 10).

Insights from qualitative interviews

To gain a deeper insight into the experiences and perceptions of the respondents regarding CP and its management, selected verbatim responses are presented below in Table 2 along with the recurring themes based on those responses.

**Table 2:** Thematic assessment of qualitative interviews along with the verbatim responses of respondents

Theme	Elaboration with representative verbatim responses
(i) Treatment initiation and antihistamines	Practitioners often start with antihistamines for immediate relief but investigate the underlying cause for long-term management. "It is very customized... we try to understand the etiology." (Dr. V)
(ii) First- vs. second-generation antihistamines	First-generation antihistamines (e.g., hydroxyzine) are used at night, while second-generation antihistamines are used during the day. "Sedative category-1 at bedtime, non-sedative in the morning." (Dr. G)
(iii) Topical adjuncts and moisturization	Topical lotions and emollients are important for managing dry skin and pruritus in combination with antihistamines. "Moisturizers with antihistamines control pruritus." (Dr. G)
(iv) Sustained-release hydroxyzine	Sustained-release hydroxyzine is favoured for better compliance, reduced pill burden, and longer symptom relief. "It reduces frequency and improves patient compliance." (Dr. G)
(v) Alternatives if antihistamines fail	If antihistamines are ineffective, steroids and anti-psychotics may be used. "Methotrexate, cyclosporin, and steroids are added if needed." (Dr. SA)
(vi) Challenges in chronic pruritus management	The main challenge faced by the majority of respondents was difficulty in diagnosing the cause of CP. "Understanding etiology is the biggest challenge." (Dr. V)
(vii) Patient reluctance and non-compliance	Another major challenge was patients' resistance to treatment due to concerns about addiction or ineffectiveness of antihistamines and their non-compliance. "Patients are reluctant to take antihistamines due to addiction concerns." (Dr. G)
(viii) Need for research and better diagnostics	More research on sustained-release formulations and better diagnostic tools were reported to be required as support by respondents. "Better diagnostic facilities would help establish etiology." (Dr. V)

## Discussion

CP is a major diagnostic and therapeutic problem with diverse causes and can have a significant impact on patients' quality of life [17]. In this study, most dermatologists reported that dry skin was the most common cause of pruritus, followed by eczema, allergies, and infections. These findings are consistent with previous studies, which highlight dry skin as a key factor in dermatoses, such as xerosis, atopic dermatitis, and psoriasis, as well as in systemic diseases, including kidney disease, liver disease, and diabetes mellitus [18,19].

CP is associated with sleep disturbances, anxiety, persistent itching, and overall decline in the quality of life, which support the findings of this study [18,20]. Furthermore, most dermatologists acknowledged the role of histamine and H1 receptors in the induction of pruritus, aligning with established evidence that histamine is a major mediator of itch. Oral antihistamines have traditionally been the cornerstone of pruritus treatment [2]. In the qualitative interview as well, all 15 dermatologists reported antihistamines as the gold standard option for the management of CP, further highlighting the role of antihistamines.

The preference for hydroxyzine, as a first-line treatment for CP observed in this study, is in line with existing literature. Previous studies have highlighted hydroxyzine, a first-generation H1 antihistamine, as one of the most commonly prescribed agents for pruritus of allergic origin due to its effectiveness, tolerability, and improvement in quality of life [21,22]. Notably, dermatologists in this study also favored hydroxyzine for its additional anxiolytic effects, a benefit supported by the European Guidelines on Chronic Pruritus, which recommends hydroxyzine as a first choice due to its antipruritic, anxiolytic, and sedative properties [23].

In prior research, a combined regimen involving sedative antihistamines like hydroxyzine at night and non-sedating second-generation antihistamines such as loratadine or cetirizine during the day was commonly employed to manage symptoms and relieve pruritus [24]. Findings in this study indicate a shift from such combination regimens towards OD evening dosing with the sustained-release formulation of hydroxyzine. This reduced dosing frequency translates into improved patient compliance, further underlining the effectiveness of sustained-release hydroxyzine in the management of CP. This is also supported by earlier studies that demonstrated the sustained-release formulation's ability to provide consistent 24-hour symptom control with an OD dose, making it particularly effective for severe acute and CP [21,25].

Moreover, while previous studies have emphasized up-dosing antihistamines to enhance efficacy in refractory cases [21], the current study revealed that 64 (31.06%) of the 206 respondents never increased the dosing frequency of hydroxyzine and 75 (36.4%) never up-dosed the sustained-release formulation for any indication. These numbers highlight the potential of sustained-release formulation to minimize the need for dosage escalation. This aligns with the trend towards simplified dosing regimens that not only maintain therapeutic effectiveness but also enhance patient adherence, a critical factor in managing chronic conditions.

Patient awareness and education regarding CP were reported as key support required by the respondents, which has also been highlighted in a previous study on the perception of CP in adults by Moosa et al. They reported that the perception of the cause was diverse in patients with CP due to the lack of provision of a clear explanation by their physicians. And this lack of explanation leads to therapeutic experimentation, alternative therapy, self-isolation, avoidance behaviors, emotional disturbance, and dermatological complications [26].

The current study included a small heterogeneous voluntary sample, thereby limiting the generalizability to a larger population. As the study relied on self-reporting, results may have been influenced by social desirability bias and recall errors, thereby potentially leading to the overestimation of "good" practices. Being a cross-sectional design, inference on the causality or changes in participants' KAP over time could not be made. Larger longitudinal studies should be undertaken in the future to overcome these limitations.

## Conclusions

Dermatologists demonstrated a good understanding of CP, particularly its pathophysiology and management. Hydroxyzine and its sustained-release formulation were recognized for therapeutic benefits, improved adherence, and favorable safety. Combination regimens with first-generation antihistamines at night and second-generation agents in the morning were common prior to the adoption of sustained-release hydroxyzine, which was considered useful for better symptom control and compliance. Overall, enhancing patient education and awareness was identified as crucial for the effective management of CP. Future research should include randomized controlled trials comparing antihistamine strategies, including sustained-release formulations, to generate stronger evidence for practice guidelines. Broader epidemiological studies should also be carried out to capture variation in prescribing practices and to validate these findings across diverse populations.

## Appendices

**Table 3:** Questionnaire for the KAP study on chronic pruritus and its management IFSI: International Forum for the Study of Itch; QOL: quality of life; OD: once daily; BID: twice daily; TDS: thrice daily; KAP: knowledge, attitudes, and practices

Demographic data
1. Name
2. Age
3. Gender
4. Place of practice (city)
5. Years of experience
6. Affiliation
7. Number of cases of chronic pruritus on a weekly basis:
(a) More than 20 cases
(b) 10-20 cases
(c) Less than 10 cases
Section 1: Knowledge About Chronic Pruritus and Its Management (Please answer the following questions by selecting the appropriate options)
1. Chronic pruritus can be defined as itching that lasts for:
(a) Less than a week
(b) >1 week but <6 weeks
(c) More than 6 weeks
2. The most common cause of chronic pruritus is:
(a) Dry skin
(b) Eczema
(c) Allergies
(d) Infections
(e) Others (please specify):
3. Atopic dermatitis is the most common cause of chronic pruritus in children.
(a) Agree
(b) Disagree
(c) Don't know
4. According to the IFSI classification of pruritus, Group I pruritus is primarily on diseased/inflamed skin.
(a) Agree
(b) Disagree
(c) Don't know
5. According to the IFSI classification of pruritus, Group II pruritus is on normal skin.
(a) Agree
(b) Disagree
(c) Don't know
6. What problem(s) do patients with chronic pruritus face?
(a) Persistent itching
(b) Impacted QOL: anxiety/mood disturbance/stress
(c) Sleep disturbance
(d) All of the above
7. Histamines and H1 receptors play a critical role in inducing pruritus.
(a) Agree
(b) Disagree
(c) Don't know
8. Oral H1 antihistamines are often considered the first line of treatment for chronic pruritus.
(a) Agree
(b) Disagree
(c) Don't know
9. Even if the origin of chronic pruritus is unknown, H1 antihistamines can be used for its management.
(a) Agree
(b) Disagree
(c) Don't know
10. In cases where chronic pruritus has more than one associated pathology, H1 antihistamines can be used as part of the treatment plan to manage itching.
(a) Agree
(b) Disagree
(c) Don't know
11. Hydroxyzine is one of the H1 oral antihistamines that can be effectively used to control and manage chronic pruritus.
(a) Agree
(b) Disagree
(c) Don't know
12. Hydroxyzine exhibits antipruritic as well as anxiolytic properties.
(a) Agree
(b) Disagree
(c) Don't know
13. The sustained-release formulation of hydroxyzine has a uniform drug plasma concentration for a prolonged period compared to hydroxyzine which reduces the dose frequency.
(a) Agree
(b) Disagree
(c) Don't know
Section 2: Attitude Towards Chronic Pruritus and Its Management (Please answer the following questions by selecting the appropriate options)
1. Chronic pruritus affects quality of life.
(a) Agree
(b) Disagree
(c) Don't know
2. Chronic pruritus causes sleep disturbances and anxiety in patients.
(a) Agree
(b) Disagree
(c) Don't know
3. Treatment plan for chronic pruritus should not only be focused on treating the underlying pathology but should also address itching and associated anxiety.
(a) Agree
(b) Disagree
(c) Don't know
4. Do you consider hydroxyzine to be safe for the management of chronic pruritus?
(a) Yes
(b) No
(c) Don't know
5. Anxiolytic properties of hydroxyzine may benefit chronic pruritus patients and improve their QOL, making it a better option for chronic pruritus management.
(a) Agree
(b) Disagree
(c) Don't know
6. Do you feel the sustained-release formulation of hydroxyzine can be beneficial in managing chronic pruritus?
(a) Yes
(b) No
(c) Don't know
7. Do you believe the sustained-release formulation of hydroxyzine has improved bioavailability compared to hydroxyzine?
(a) Agree
(b) Disagree
(c) Don't know
8. Do you feel the sustained-release formulation of hydroxyzine may reduce the need for up-dosing?
(a) Yes
(b) No
(c) Don't know
9. Do you feel the lower frequency of dosing with the sustained-release formulation of hydroxyzine leads to better patient tolerability and compliance?
(a) Agree
(b) Disagree
(c) Don't know
Section 3: Practices Related to Chronic Pruritus and Its Management (Please answer the following questions by selecting the appropriate options)
1. In how many cases do you prescribe H1 antihistamines to manage chronic pruritus?
(a) All cases with a few exceptions
(b) >50%
(c) <50%
(d) None
2. How often do you consider hydroxyzine as a first-line treatment option for chronic pruritus?
(a) Rarely or never
(b) Occasionally
(c) Frequently
(d) Always
3. Which of the following drugs do you prescribe for the management of chronic pruritus?
(a) Hydroxyzine
(b) Sustained-release hydroxyzine
(c) Levocetirizine
(d) Fexofenadine
(e) Loratadine
(f) Cetirizine
(g) Bilastine
(h) Others (please specify):
4. What factors would influence your decision to prescribe hydroxyzine over other antihistamines for chronic pruritus?
(a) Patient's age and comorbidities
(b) Severity and duration of pruritus
(c) Presence of specific underlying conditions
(d) Patient's preference and treatment history
(e) All of the above
(f) None, do not prescribe hydroxyzine
5. In your clinical experience, how effective is hydroxyzine in managing chronic pruritus?
(a) Very effective
(b) Moderately effective
(c) Ineffective
(d) Don't prescribe hydroxyzine
6. What is the tolerability of hydroxyzine compared to other H1 antihistamines in your practice?
(a) Significantly better tolerated
(b) Slightly better tolerated
(c) Similar tolerability
(d) Slightly less tolerated
(e) Significantly less tolerated
7. In your practice, what dosing schedule do you use while prescribing hydroxyzine for the management of chronic pruritus?
(a) OD
(b) BID
(c) TDS
(d) Don't prescribe hydroxyzine
8. Do you prescribe the sustained-release formulation of hydroxyzine in your practice?
(a) Yes
(b) No
The next set of questions is for the respondents who respond with "Yes" to Question 8
9. What dosing schedule do you follow while prescribing the sustained-release formulation of hydroxyzine?
(a) OD
(b) BID
(c) TDS
(d) Others (please specify):
10. In what conditions do you prescribe more than once a day dosing of hydroxyzine?
(a) Scabies
(b) Prurigo nodularis
(c) Senile pruritus
(d) Others (please specify/elaborate):
11. In your practice before using the sustained-release formulation of hydroxyzine, what treatment option(s) do you use for the management of chronic pruritus?
(a) Night-time sedative antihistamine+day-time non-sedative antihistamine
(b) Up-dosing of the same antihistamine
(c) Multiple doses of antihistamine
(d) Others (please specify):
12. Do you find the sustained-release formulation of hydroxyzine to be more effective than the non-sustained-release formulation?
(a) Yes
(b) No
(c) Don't know
13. Do you find the sustained-release formulation of hydroxyzine to be less sedative than the non-sustained-release formulation?
(a) Yes
(b) No
(c) Don't know
14. Which benefit of the sustained-release formulation of hydroxyzine do you find most useful in treating chronic pruritus and related indications?
(a) 24-hour symptom/pruritus control
(b) Convenience of OD dosage
(c) Less sedative
(d) Anxiolytic benefit
(e) All of the above
15. In what chronic pruritus indications do you up-dose the sustained-release formulation of hydroxyzine from OD?
(a) Scabies
(b) Prurigo nodularis
(c) Senile pruritus
(d) Others (please specify/elaborate):
16. Do you use the sustained-release formulation of hydroxyzine for the management of acute pruritus as well?
(a) Yes
(b) No
Set of questions below to be asked to respondents who respond with "No" to Question 8
9. What current treatment regimen do you follow for the management of chronic pruritus?
(a) Hydroxyzine
(b) Levocetirizine
(c) Fexofenadine
(d) Loratadine
(e) Cetirizine
(f) Bilastine
(g) Others (please specify):
10. Which molecule/drug would you replace from your current regimen to prescribe the sustained-release formulation of hydroxyzine for the management of chronic pruritus?
(a) Hydroxyzine
(b) Levocetirizine
(c) Fexofenadine
(d) Loratadine
(e) Cetirizine
(f) Bilastine
(g) Others (please specify):
Section 4: Qualitative Questions for Face-to-Face Interviews
1. Please share any additional insights or experiences you have regarding the use of antihistamines for chronic pruritus management.
2. Please share your preferred treatment plan for chronic pruritus patients.
3. Place of the sustained-release formulation of hydroxyzine in your clinical practice?
4. What are the challenges you face in managing chronic pruritus in your practice?
5. What resources or support would you find helpful in improving your approach to chronic pruritus management?
